# The Experimental and Clinical Aspects of Beta Cell Function and Its Underlying Mechanism

**DOI:** 10.1155/2014/659149

**Published:** 2014-06-18

**Authors:** Yanbing Li, Li Chen, Chen Wang, Dongqi Tang

**Affiliations:** ^1^Department of Endocrinology, The First Affiliated Hospital, Sun Yat-Sen University, Guangzhou 510080, China; ^2^Department of Endocrinology, Qilu Hospital of Shandong University, Jinan 250012, China; ^3^Department of Endocrinology and Metabolism, Shanghai Jiao Tong University Affiliated Sixth People's Hospital, Shanghai 200233, China; ^4^Department of Pathology, Immunology, and Laboratory Medicine, University of Florida, Gainesville, FL 32611, USA

The development of diabetes mellitus is the interplay between insulin secretion and insulin resistance, while insufficient compensatory beta cell function plays a major role during the natural progression of the disease. In the past decades, a lot of work had been carried out to investigate the initiation and regulation of beta cell dysfunction, yet the underlying mechanism is largely unknown. In this issue, 11 interesting papers are compiled to discuss from experimental and clinical aspects the mechanism of beta cell dysfunction in the development of diabetes.

MicroRNAs (miRNAs) are small noncoding 18–25 nucleotides that bind to the complementary 3′UTR regions of target mRNAs and function in transcriptional and posttranscriptional regulation of gene expression. In recent years, miRNAs were reported to regulate several metabolic pathways including insulin secretion, cholesterol biosynthesis, carbohydrate, and lipid metabolism. In this issue, 3 papers touched upon the regulatory effect of some miRNAs on beta cell function from different points of view. Dr. X. Chang et al. reported that the mean level of miR-375 methylation was significantly lower in T2DM patients from Kazak population than Han population, which might partly explain from genetic background that even though Kazak population clusters more risk factors for T2DM, prevalence rate of T2DM is 6 times less than that of the Han population in the same region. Q. Zhang et al. showed that 8-week treatment of Tianmai Xiaoke Tablet, a chromium picolinate hypoglycemic agent, in diabetic rat significantly upregulated the expression of multiple miRNAs such as miR-375 and miR-30d, which might be part of its effect on improving glucose control. X. Lin et al. investigated the direct suppression of Bcl-2 by miR-34a, which might account for palmitate-induced apoptosis in MIN6 cell, the latter of which is believed to be the most important mechanism of beta cell dysfunction. Another 2 manuscripts also discussed beta cell apoptosis. Dr. L. Zhou et al. examined the 3 signaling pathways of MAPKs in INS-1 cells treated with glucolipotoxicity conditions and concluded that P38 might be involved in the regulation of beta cell apoptosis through phosphorylation of IRS-2. Z. Zhang et al. simulated intermittent high glucose situation* in vitro* and investigated its effect on beta cell apoptosis. In another paper, the attenuating effect of metformin against high glucose-induced suppression of cell proliferation and osteogenic-related gene expression in osteoblast was investigated by X. Shao et al.

Three papers in this issue presented with unique clinical picture of diabetes in the Chinese population. W. Tang et al. investigate the relationship between serum uric acid and residual beta cell function in 1021 T2DM patients. They concluded that patients with higher serum uric acid had greater insulin secretion at the early stage, but their residual beta cell function decayed more rapidly. Y. Ma et al. compared newly diagnosed T2DM patients with or without hyperlipidemia and found that the former were younger and had worse beta cell function. H. Lu et al. reported that ketosis onset type 2 diabetic patients had better beta cell function and were more insulin resistant.

Two manuscripts brought new exploration in the treatment of type 1 diabetes. Dr. W. Li et al. revealed that, apart from higher density of insulin-producing beta cells, small islets transplantation expressed less angiotensin and more angiotrophic VEGF-A, which might be beneficial for the facilitation of microcirculation and revascularization in small islets. H. Luo et al. presented with a novel protocol that reprogrammed primary hepatocytes into functional insulin-producing cells using multicistronic vectors carrying Pdx1, Ngn3, and MafA. These cells activated multiple beta cell gene expression, synthesized and stored considerable amounts of insulin, and released the hormone in a glucose-regulated manner* in vitro*.

We hope to bring about extensive concern and energetic discussion about beta cell function from experimental as well as clinical aspects. We wish that our readers enjoy this special issue.

*Yanbing Li* *Yanbing Li* *Li Chen* *Li Chen* *Chen Wang* *Chen Wang* *Dongqi Tang* *Dongqi Tang*

